# Prolonged disengagement from distractors near the hands

**DOI:** 10.3389/fpsyg.2013.00533

**Published:** 2013-08-15

**Authors:** Daniel B. Vatterott, Shaun P. Vecera

**Affiliations:** Department of Psychology, University of IowaIowa City, IA, USA

**Keywords:** visual attention, visual processing near hands, attentional capture, attentional control, hand position

## Abstract

Because items near our hands are often more important than items far from our hands, the brain processes visual items near our hands differently than items far from our hands. Multiple experiments have attributed this processing difference to spatial attention, but the exact mechanism behind how spatial attention near our hands changes is still under investigation. The current experiments sought to differentiate between two of the proposed mechanisms: a prioritization of the space near the hands and a prolonged disengagement of spatial attention near the hands. To differentiate between these two accounts, we used the additional singleton paradigm in which observers searched for a shape singleton among homogenously shaped distractors. On half the trials, one of the distractors was a different color. Both the prioritization and disengagement accounts predict differently colored distractors near the hands will slow target responses more than differently colored distractors far from the hands, but the prioritization account also predicts faster responses to targets near the hands than far from the hands. The disengagement account does not make this prediction, because attention does not need to be disengaged when the target appears near the hand. We found support for the disengagement account: Salient distractors near the hands slowed responses more than those far from the hands, yet observers did not respond faster to targets near the hands.

Interacting with objects in the real world requires several cognitive and perceptual processes to be integrated. Consider the simple example of picking up your coffee cup: This seemingly simple act requires (1) coordination between a visual representation of the object's shape and spatial location (necessary for basic visual perception), (2) selective (attentional) processing to the object (necessary to minimize interference from other objects), and (3) the current state and position of one's body (necessary for planning a movement). Although interacting with objects may require other perceptual-cognitive operations, both spatial attention and body position are critical for the initial localization of and interaction with objects in everyday situations. Understanding how the body guides and interacts with visual inputs in directing spatial attention is important for accounts of real-world behavior.

Bimodal neurons in premotor and parietal cortex have visual receptive fields that surround part of the body (e.g., the hand), and this visual receptive field shifts in space as the body part moves (Graziano and Gross, 1993, 1998). One potential behavioral consequence of these neurons is that body position affects cross modal spatial attention as well as visual inputs. Recent behavioral studies have demonstrated that the body indeed influences spatial attention, both in neurologically normal participants (for a review, see Brockmole et al., 2013) and brain-damaged patients with attentional disruptions (di Pellegrino and Frassinetti, 2000; Schendel and Robertson, 2006). Although hand position affects spatial attention, the mechanisms of this influence are still the focus of active study. Some accounts propose that the body prioritizes attention in the direction of the hand (Reed et al., 2006), whereas others hypothesize that hand position influences perceptual-level processing (Cosman and Vecera, 2010). Further, some accounts argue that hand position influences specific attentional operations, such as attentional disengagement (Abrams et al., 2008), or biases processing toward certain types of information, such as high temporal frequencies (Gozli et al., 2012).

The current experiment aims to distinguish whether the body prioritizes attention toward the hand or slows the disengagement from items near the hand. To differentiate between these two accounts, we turned to the additional singleton paradigm (Theeuwes, 1992; see Theeuwes, 2010, for a review). In the additional singleton paradigm, observers look for a shape singleton target among homogenous distractors (e.g., a circle among diamonds) and respond to an irrelevant feature of the target (orientation of a line within the target). Importantly, on half the trials, one of the distractors is a different color (i.e., a color singleton). The target is never a color singleton, so there is no reason to attend to the color singleton, so if attentional control is perfectly tuned for an observer's goals, then observers should devote no processing to this color singleton distractor. Interestingly, researchers consistently find observers respond slower to the target when the color singleton is present than when it is absent (Theeuwes, 2010), and these slower response times reflect processing of the color singleton. Thus, to differentiate whether hand position modulates attentional prioritization or disengagement, we used the additional singleton paradigm in Experiment 1A. We reasoned that both the attentional prioritization and disengagement accounts predict observers will respond to the target slower when the color singleton appears near the hand than when it appears far from the hand because items near the hand are either prioritized or disengagement from these items is prolonged. Critically, the attentional prioritization account also predicts that because the space near the hands is prioritized, observers will respond faster to targets near the hands than far, but the disengagement account does not make this prediction.

It is possible hand position will have a small effect on task performance because the additional singleton paradigm traditionally uses consistently defined target and distractors, which encourages observers to guide attention based on the task features (Lamy et al., 2006). That is, observers might rely on a well-learned target template for the circle target, thereby minimizing the hand's overall influence. Thus, to fully evaluate the target prioritization account, it is critical to prevent attentional guidance by features. To discourage observers from guiding search based on target and distractor features, we used the mixed version of the additional singleton paradigm in Experiment 1B (Pinto et al., 2005). In this version of the task, the target/distractor identities and object colors change from trial to trial. For example, on one trial, the target could be defined as a circle among diamonds and on the next it could be a diamond among circles. Additionally, the color of the target changes from trial to trial. These changes minimize the opportunity for observers to guide attention based on a target template (i.e., a specific shape or color feature) other than a singleton search mode (Pashler, 1988).

To investigate whether attention is prioritized near the hands or attention is slower to disengage from items near the hands, half the observers completed the additional singleton paradigm with either their left or right hand near the screen. The other half of participants completed the mixed version of the additional singleton paradigm with either their left or right hand near the screen. Slower responses to the target when the color singleton was near the hand than far from the hand will serve as a manipulation check because both accounts predict this. Critically, if attention is prioritized to items near the hands, then observers should be faster to respond to targets near the hands than far, but if observers are slower to disengage from items near the hands, then observers will not respond faster to items near the hands than far.

## Methods

### Observers

Thirty-two undergraduate students from the University of Iowa participated to fulfill a course requirement. Sixteen participated in Experiment 1A and sixteen participated in Experiment 1B. All observers reported normal or corrected-to-normal vision.

### Stimuli and procedure

A Mac Mini computer with a 17-in CRT monitor presented stimuli and collected response through MATLAB and the Psychophysics Toolbox (Brainard, 1997). Eight stimuli were presented around an imaginary circle centered on the screen with a radius of 6°. The stimuli consisted of seven diamonds and one circle. The stimuli were each approximately 2.8 × 2.8°. Each item contained either a gray vertical or horizontal line. The lines measured 1.5 × 0.3°. In Experiment 1A, all the items were green (RGB 20 210 5) except on half the trials, one of the diamonds was red (RGB 255 0 0). In Experiment 1B, the color (red or green) and shape (circle or diamond) of the target was chosen randomly on each trial. A white fixation dot was presented at the center of the screen and measured 0.6 × 0.6°. Additionally, two white dots (0.6 × 0.6°) were presented on the left and right sides of the screen. These dots indicated where observers' hands should place their hands.

The target appeared equally often at any of the eight possible target positions. Observers responded to the orientation of the gray line within the target. Observers responded with a left pedal if the target contained a vertical line and they responded with a right foot pedal if the target contained a horizontal line. Half the trials contained a target with a horizontal line and half the trials contained a target with a vertical line. A color singleton distractor was present on half the trials. The color singleton distractor appeared randomly at one of the seven positions not already occupied by the target.

On half the blocks the observers held their right hand up with their middle finger abutting the monitor. The palm of their hand faced toward the search array. On the other half of the blocks, observers held their left hand near the monitor. Observers' arms were supported by armrests to prevent fatigue. The order of which hand was initially held up to the monitor was counterbalanced across observers. The blocks were 28 trials long and each experimental session consisted of 896 trials. Observers were given a self-paced break at the end of each block. Finally, to keep the displays as visually balanced as possible, a visual anchor abutting the monitor was always presented opposite to the raised hand.

**Figure 1:**
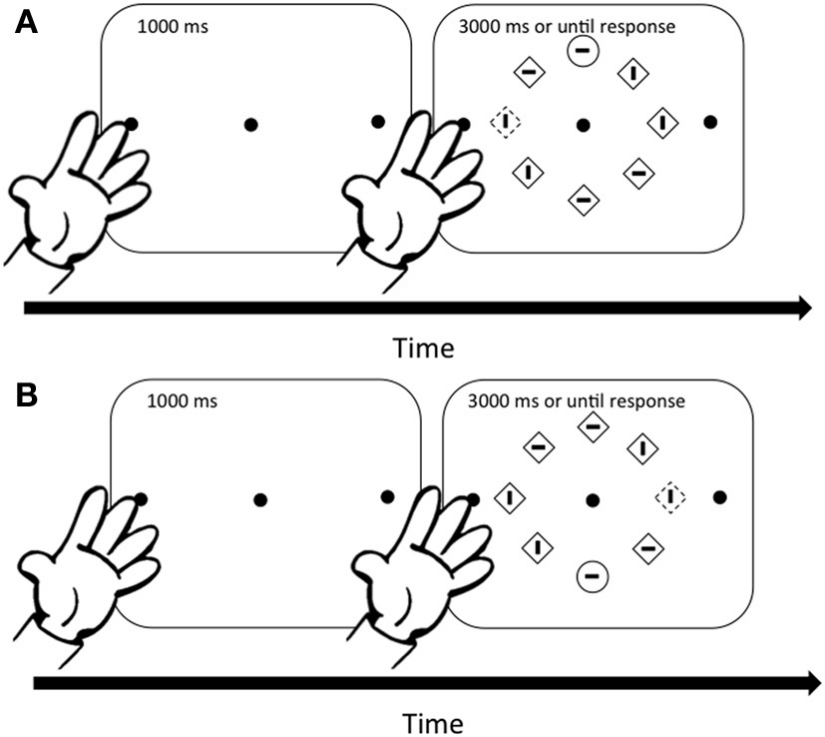
**Sequence of events for Experiments 1A,B**. A fixation dot preceded each search display by 1000-ms. The search display remained on the screen for 3000-ms or until response. Color singletons appeared on 50% of the trails. In **(A)** the color singleton appears on the horizontal meridian near the hand and the target appears on the vertical meridian. In **(B)**, the color singleton appears on the horizontal meridian far from the hand and the target appears on the vertical meridian.

Each trial started with the presentation of a fixation dot and the two dots indicating hand placement for a second. Following this, the search array was presented for 3 s or until response (see Figure 1). If observers took more than 3 s to respond, the trial was scored as incorrect and observers were encouraged to respond faster. Observers were instructed to maintain fixation and to respond as quickly and accurately as possible. Observers completed four practice blocks of trials (two with each hand up) before the experimental session.

## Results and discussion

**Figure 2:**
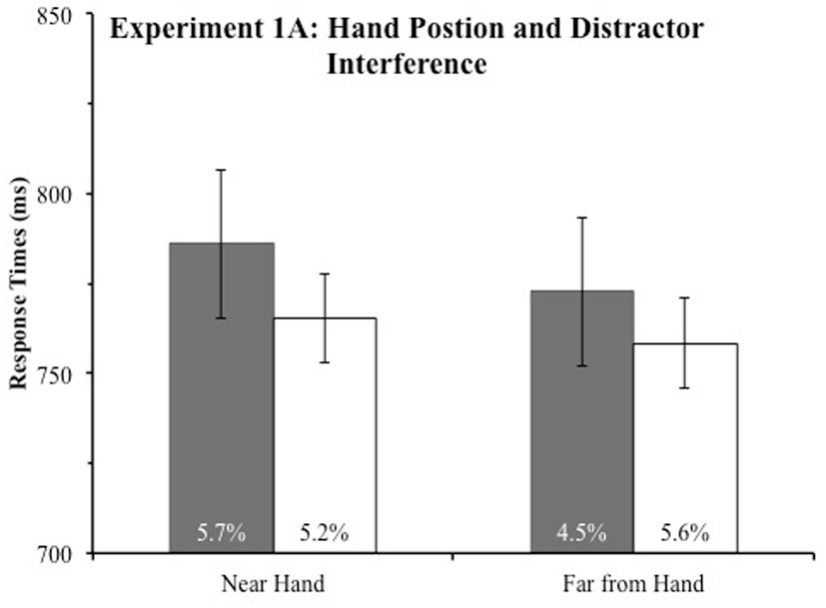
**Experiment 1A response times (in ms) as a function of item near hand (target or distractor) and distance from hand (near the hand or far from the hand)**. The error rates of each condition are reported in the base of the bars. Error bars represent 95% within-subject confidence intervals (Loftus and Masson, 1994).

**Figure 3:**
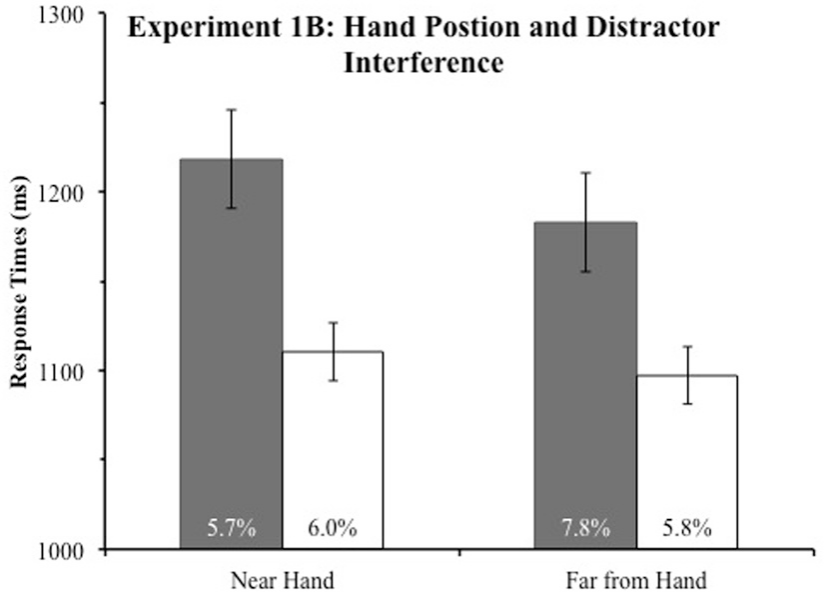
**Experiment 1B response times (in ms) as a function of item near hand (target or distractor) and distance from hand (near the hand or far from the hand)**. The error rates of each condition are reported in the base of the bars. Error bars represent 95% within-subject confidence intervals (Loftus and Masson, 1994).

Incorrect responses and RTs more than 2.5 standard deviations from an observers' condition mean were excluded from the analysis. This eliminated 1.6% of the data in Experiment 1A and 1.1% of the data in Experiment 1B. We submitted mean RTs to a mixed ANOVA with the within subject factors item near hand (Target or Distractor) and distance from hand (Near or Far). Experiment (1A or 1B) was a between subjects factor. To prevent any interference due to target and color singleton proximity (Mounts, 2000a,b), RTs in this analysis only included trials in which the target was present at one of the two positions on the vertical meridian and the color singleton was present in one of the two positions on the horizontal meridian when evaluating the effect of hand position on distractor processing and vice versa when evaluating the effect of hand position on target processing. Slower responses when the distractor was on the horizontal meridian than when the target was on the horizontal meridian drove a main effect of the item near hand factor, *F*_(1, 30)_ = 31.21, *p* < 0.001. As expected, the item near hand factor interacted with the experiment factor, *F*_(1, 30)_ = 15.05, *p* = 0.001, because, as depicted in Figures 2, 3, color singletons slowed responses more in Experiment 1B (mixed additional singleton design) than in Experiment 1A (fixed additional singleton design). Observers responded slower when the target or distractor appeared near the hand, *F*_(1, 30)_ = 3.83, *p* = 0.06, demonstrating that the hand had an effect. Slower responses to the target when the distractor is near the hand than far from the hand (depicted in Figures 2, 3) likely drove this effect. The distance from the hand factor did not interact with the experiment factor, *F*_(1, 30)_ = 0.65, *p* > 0.42, demonstrating that the hand had the same effect in the two experiments.

Interestingly, suggesting that the observers were slower to respond both when the target was near the hand and when the distractor was near the hand, the item near hand factor (target or distractor) and the distance from the hand factors did not interact, *F*_(1, 30)_ = 0.5, *p* > 0.47. The three way interaction between item near hand, distance from hand, and experiment also failed to reach significance, *F*_(1, 30)_ = 0.16, *p* > 0.68. Although the non-significance of these interactions suggests hand position did not speed responses to items near the target, because this is a central question of our study, we conducted follow up analyses to investigate differences in RTs to targets near and far from the hands in each experiment.

To evaluate the effect of hand position on target processing, we compared RTs when the target was on the horizontal meridian and the distractor, when present, was on the vertical meridian. Thus, we conducted a *t*-test comparing mean RTs to targets on the horizontal meridian near and far from the hand. Inconsistent with the prioritization account, in Experiment 1A, we found RTs to the target were no faster when the target was near the hand (765 ms) than far (758 ms), *t*_(15)_ = 0.60, *p* > 0.55. It is possible this *t*-test failed to find a difference because hand position has a different effect on target processing when the color singleton is present than absent, so we performed two additional *t*-tests on RTs from Experiment 1A, one comparing RTs to targets near and far from the hand when the color singleton was absent, *t*_(15)_ = 0.51, *p* > 0.61, and another when the color singleton was present, *t*_(15)_ = 0.37, *p* > 0.72. Thus, these tests falsify the prioritization account and lend tentative support to the disengagement account of hand position's effect on cognition.

To evaluate if hand position affected responses to the target in Experiment 1B, we ran the same *t*-tests as in Experiment 1A. Again, the results falsified the prioritization account since RTs to the target did not differ when the color singleton was near (1097 ms) and far from the hand (1110 ms), *t*_(15)_ = 0.87, *p* > 0.4. Again, responses to targets near and far from the hand did not depend on the presence or absence of the color singleton because these *t*-tests also failed to reach significance [Absent: *t*_(15)_ = 0.004, *p* > 0.99; Present: *t*_(15)_ = 0.76, *p* > 0.45]. Thus, our experiments demonstrate that observers were no faster to respond to targets near their hands, which is inconsistent with an attentional prioritization of items near the hand account and lends tentative support to the slowed disengagement from items near the hands account.

We repeated all RTs analyses with arcsine-transformed error rates. The mixed ANOVA and planned follow-up comparisons all failed to reach significance. We suspect these comparisons failed to reach significance because accuracy values were so close to ceiling. Importantly, the lack of significant values also demonstrates that the RT differences in this experiment cannot be explained by a speed accuracy trade-off.

## General discussion

The mechanism behind hand position's effect on visual attention is an open question and the current experiment sought to differentiate between the prioritization of items near the hands (Reed et al., 2006) and prolonged disengagement from items near the hands accounts (Abrams et al., 2008). To differentiate between these accounts, we used the additional singleton paradigm because both accounts predicted greater slowing from color singletons near the hands than far from the hands. Importantly, the prioritization account predicts faster responses to targets near the hands while the disengagement account does not. We rejected the prioritization account and we tentatively support the disengagement account because neither experiment 1A nor experiment 1B found faster responses to targets near than far from the hands while the two experiments did find slower RTs when a distractor or target appeared near the hands than far from the hands.

Our experiments did not seek to evaluate the perceptual-level processing (Cosman and Vecera, 2010) and bias toward high temporal frequency accounts of hand position (Gozli et al., 2012). These accounts are still plausible especially since it is reasonable to speculate that hand position has multiple different effects along the processing stream. Future experiments should evaluate these different accounts of the mechanism behind hand position effects.

One additional explanation of our data is that the color singleton distractors slowed response times not because they captured attention, but because color singletons require more pre-attentional processing (i.e., a filtering cost; Folk and Remington, 1998). For instance, it is possible that items near the hand take longer to process than items far from the hands, but we find this hypothesis unlikely for a number of reasons. First, ERP evidence supports the attentional capture account of the additional singleton paradigm (Hickey et al., 2006). Second, we believe it is unlikely the cognitive system is designed to process items near the hands slower than items far from the hands because items near the hands are likely important, and, if anything, should be processed faster.

One interesting question is why Reed et al. (2006) found faster detection of targets near the hands, but we did not find faster responses to targets near the hands. It is possible that hand position is simply weighted like any other input to the attentional mechanism (Wolfe, 1994) and that when feature values are important, feature values are more heavily weighted and hand position is less weighted. Thus, in an experiment like Reed and colleagues' it is possible that the sparse displays provided so little information that hand position was more heavily weighted (i.e., prioritized). Basically, we propose that whether the space near the hands is prioritized may interact with the amount of information observers have to complete the rest of the task. When observers have little information to help them complete a task, such as in a Posner cuing task, the space near the hands is prioritized, but when observers are able to guide task performance with information such as target features, the space near the hands is not prioritized. We are currently running experiments to evaluate this possibility.

Because of hand position's importance to many daily activities, spatial attention changes near the hands. The current experiments sought to evaluate between two accounts of exactly how spatial attention changes near the hands. The first account is that the space near the hands is prioritized and the second account is that observers are slower to disengage from items appearing near their hands. We failed to find support for the prioritization account, so we tentatively support the disengagement account that observers are slower to disengage from items near their hands. Thus, our experiments suggest that the hands might not always change the processing of items near the hands and instead hand position might extend the processing of these potentially important items.

### Conflict of interest statement

The authors declare that the research was conducted in the absence of any commercial or financial relationships that could be construed as a potential conflict of interest.
